# Potentiometric determination of mebeverine hydrochloride antispasmodic drug based on molecular docking with different ionophores host–guest inclusion as a theoretical study

**DOI:** 10.1039/d2ra06127a

**Published:** 2023-01-04

**Authors:** Ahmed M. Abdel-Raoof, Manal M. Fouad, Noha S. Rashed, Noha Y. Z. Hosni, Ahmed Elsonbaty, Ashraf Abdel-Fattah

**Affiliations:** a Analytical Chemistry Department, Faculty of Pharmacy (Boys), Al-Azhar University Cairo 11751 Egypt ahmedmeetyazeed79@yahoo.com Ashraf.abdelfattah@hotmail.com; b Pharmaceutical Analytical Chemistry Department, Faculty of Pharmacy (Girls), Al-Azhar University Nasr City Cairo Egypt; c Pharmaceutical Analytical Chemistry Department, Faculty of Pharmacy, October University for Modern Sciences and Arts (MSA) Giza 11787 Egypt; d Pharmaceutical Chemistry Department, Faculty of Pharmacy, Egyptian Russian University Badr 11829 City Cairo Egypt

## Abstract

The scientific community has continued to pay particular attention to potentiometric sensors based on ion-selective membrane sensors as an energy-efficient, easy-to-use method suitable for microfabrication. To this end, potentiometric ion-selective sensors were used as an alternative green analytical instrument. Three distinct sensors relying on various ionophores were built and assessed. A cationic exchanger, tetra phenyl borate, was used in the polyvinyl chloride polymeric plasticized matrix using di octyl phthalate, where α, β, and γ cyclodextrins were utilized as ionophores. A comparative potentiometric analysis was carried out using three ion-selective sensor designs: α, β, and γ cyclodextrins sensors. β-Cyclodextrin significantly reduced the detection limit and improved the discriminative performance of mebeverine hydrochloride (MBV) in the pharmaceutical dosage form over α- and γ-cyclodextrins in the presence of other interfering chemicals. Additionally, a significant connection was made between the practical perspective and a theoretical investigation based on computational research. Nernstian potentiometric results for the optimum sensor were obtained for MBV in the range of concentrations 1.0 × 10^−2^ to 1.0 × 10^−6^ M, its slope was −58.70 ± 0.12 mV per decade with lower detection limits 4.50 × 10^−7^ M. This computational molecular docking investigation clarified that the binding sites and modes were in good agreement with the experiment results. This investigation was applied to expect the interaction between MBV and the proposed sensors to ensure which ionophores were the best for MBV.

## Abstract 2

A novel ion selective sensor for detecting MBV in pharmaceutical formulations has been fabricated supported by computational molecular modeling methods.

## Graphical abstract

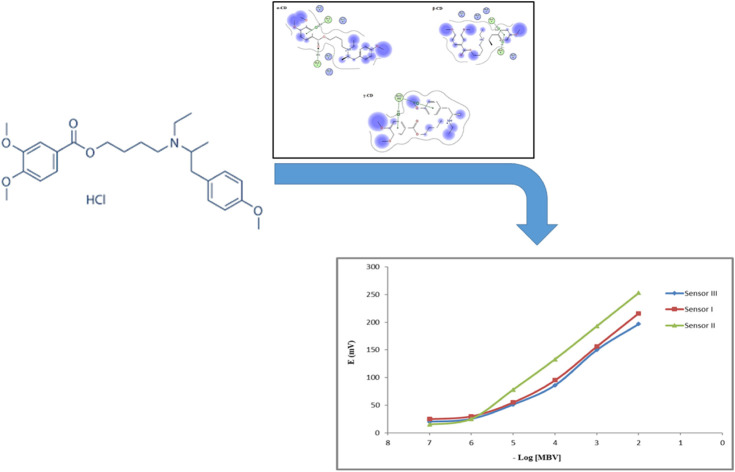

## 1. Introduction

Mebeverine hydrochloride (MBV) is frequently used as an antispasmodic drug to treat muscular spasms, and gastrointestinal spasms following cholecystectomy, as well as to relieve uncomfortable stomach cramps in people with irritable bowel syndrome and other diseases.^1^

A comprehensive study of the literature revealed the dominance of analytical methods for MBV, including spectrophotometric,^2–11^ HPLC,^12–18^ fluorometric,^19,20^ and electrochemical methods.^21–27^ Although these methods provided a high level of specificity, they are unable to apply rapid *in situ* detection of MBV. Moreover, these previously described methods still had several limitations, such as the need to use a lot of expensive, hazardous organic reagents that would hurt the environment. In addition, employing advanced tools made practically all of these procedures time-consuming. These difficulties mirrored the chemist's concerns about using potentiometric, environmentally green analytical methods. Its benefits include being a straightforward, delicate, affordable, and reliable technology that enables its use in various study fields. Ion-selective electrodes are more selective, more quickly responsive, and portable, making the design and assembly more straightforward. Moreover, several investigations revealed that when ionophore ion-selective sensors were used, selectiveness, responsiveness, and the limited detection of electrode sensors were significantly enhanced when contrasted to ion pairs relying upon electrodes.

The application and using potentiometric ion-selective electrodes to evaluate various medications employing different modifiers of cyclodextrins (CD) based on the host–guest inclusion process has been the subject of several investigations.^28,29^ The development of non-covalent interactions between the guest and host is necessary to form the inclusion complexes. The recognition and inclusion of molecular complexation (MC) are essential for supramolecular chemistry and the host–guest method. Cyclodextrins (CD), which include alpha-CD, beta-CD, and gamma-CD, are a kind of MC agent. They are toroidal structures with an internal cavity that is hydrophobic and non-polar, and an outer surface containing lyophobic and hydroxyl groups. They quickly created water-soluble inclusion complexes with a variety of poorly soluble chemicals because of this distinctive structure.

The uniqueness of the current study lies in the compromise of the advantages of both, the ion exchanger and CD ionophore as reflected in the predicted electrode performance. This research aims to create a novel CD-modulated ion-selective sensor for detecting MBV in pharmaceutical formulations. Additionally, computational molecular modeling methods were employed to clarify the properties of CD-MBV complexes and demonstrate the molecular level selectivity of the sensors.

## 2. Experimental

### 2.1. Materials

Mebeverine HCl (MBV) with a purity rating of 98.69% ± 0.54 was provided by EPICO pharmaceutical industries (10th of Ramadan, Egypt). Tributyl phosphate (TBP), tetrahydrofuran (THF), sodium tetraphenylborate, benzyl acetate (BA), di-octyl phthalate (DOP), alpha, beta, and gamma-cyclodextrin (CD), and polyvinyl chloride (PVC) were obtained from Sigma-Aldrich (Germany). We received the following chemicals from Prolabo (Paris, France): MgCl_2_, NaCl, sucrose, glucose, BaCl_2_, lactose, KCl, urea, NiCl_2_·6H_2_O, glycine, and NH_4_Cl. HCl, orthophosphoric acid, NaOH, and glacial acetic acid were purchased from El-Nasr Company (Cairo, Egypt). The water utilized throughout the whole procedure was double distilled.

Pharmaceutical formulation: Colospasmin® forte tablets containing 135 mg MBV were purchased from a local store.

### 2.2. Instrumentation

A digital pH 3505 (Jenway-USA) digital ion analyzer with a double-junction Ag/AgCl reference electrode was employed in conjunction with PVC-MBV ion selective electrodes to perform all the potentiometric mensuration. The pH was adjusted using a Jenway pH glass electrode. All potentiometric evaluations were performed at ambient temperature. The molecular operating environment 2019.10 software was used to perform a computational study.

### 2.3. Standard solutions

The preparation of 0.01 M MBV stock standard solution was achieved by dissolving 466 mg of the MBV standard powder in a 100 mL volumetric flask comprising 50 mL of Britton–Robinson buffer (0.04 M) of pH 7. A serial dilution was accomplished to prepare working solutions of MBV in the range of 1 × 10^−6^ to 1 × 10^−3^ M utilizing the same solvent.

### 2.4. Procedures

#### 2.4.1. Ion-pair preparation (IP)

IP association is considered the sensing component used in the suggested electrode, which was formed by the interaction of Na-TPB and MBV. The previously published research^25^ demonstrated that MBV and Na-TPB were applied as a complex in the ratio of 1 : 1. As a result, the MBV-TPB ion-pair interaction was formed by adding 60 mL of MBV solution to 60 mL Na-TPB, both having the same concentration of 1 × 10^−2^ M. The resulting residue was allowed to be left nocturnally to achieve maximum coagulation before being filtered and rinsed with double distilled water. The coagulated part was left for 24 h at ambient temperature to ensure its complete dryness before its usage.

#### 2.4.2. Preparation of MBV-ionophore-modified PVC membranes

The desired three membranes were prepared by adding 190 mg of PVC portion-wise to each glass Petri dish of 5 cm size in diameter, comprising 296 mg of DOP as a plasticizer, 8 mg of IP, and 5 mL of THF. Then, the membrane ingredients were mixed very well using a glass rod before separately adding 10 mg of α, β, and γ CD. After that, the resulting mixtures were transferred to stoppered vessels for complete mixing for 5 min utilizing a magnetic stirrer. Finally, each mixed solution was returned to the Petri dish, finally enveloped, and left overnight at ambient temperature, ensuring complete evaporation of THF.

#### 2.4.3. Electrode assembly

Three different electrodes were assembled by cutting three discs (with a diameter of 10 mm for each one). The previously prepared membranes were affixed using THF to a PVC tip attached to the end part of the glass electrode. Then, the electrode was left for a fixed time to ensure the complete attachment of the membrane to the PVC part of the electrode. After that, each electrode was filled with equal volumes of 1 × 10^−2^ M of MBV solution and KCl solution. Finally, conditioning of each electrode by soaking them in 1 × 10^−2^ M of MBV solution for about 24 h was performed. A wire with a diameter of 1 mm and made of Ag/AgCl served as the inner reference electrode.

#### 2.4.4. Pharmaceutical MBV preparation

Ten Colospasmin® forte tablets comprising 135 mg of MVB were weighted precisely, then coarsely crushed. An accurately pulverized portion of powder equivalent to one tablet was firmly transmitted into a 100 mL volumetric flask with a 60 mL Britton–Robinson buffer of pH 7. After sonicating for 10 min, the final concentration of 2.89 × 10^−3^ M of MBV was prepared by adjusting the solution volume using the same solvent to the flask mark. The suggested sensor, linked to the reference electrode, was used to record the potential, and the related regression equation was used to compute the concentration of MBV.

#### 2.4.5. Calibration curves

Each electrode was tested by dipping in 30 mL of MBV solution (1 × 10^−6^ to 1 × 10^−2^ M) with the Ag/AgCl reference electrode. After stabilization, the potential established was registered, and then the relationship between the generated potential and −log MBV concentration was graphed. The resulting regression equation was used to calculate the unknown concentrations.

#### 2.4.6. Molecular docking (MD)

The results of experimental studies were confirmed using the MOE software through a molecular docking investigation for MBV with ionophore inclusion complexes. The MD process was performed using the CD-ionophore as a selective receptor for the drug acting as a ligand. Each one of the CD-ionophore was extracted from the protein data bank (PDB) in the form of a 3D crystal structure. α-CD was isolated from the SusD protein of bacteroides with the code 3CK7, while the 3D crystal structure of the β-CD was presented in a complex with the β-amylase enzyme. Under the designation 5E70, the γ-CD crystal structure was derived from the *E. coli* branching enzyme. The structure of MBV was constructed using the ChemDraw software. Then, the Quick Prep technique for MOE was used to add hydrogen atoms to MBV and reduce its structure. Finally, by applying the correct force field, the energy was reduced. The database of conformational search results for MBV was used to employ the docking process using the MOE docking methodology at each CD molecule as a receptor. For each ligand conformation location, the triangle matcher method was used, and conformation ordering was performed using the London G scoring algorithm. Finally, the positions were categorized based on their docking assessments, and the most convenient energy position was chosen.^27^

## 3. Results and discussion

### 3.1. Optimization of the composition of the sensor

Ion-selective electrodes' selectivity, linearity, and sensitivity are influenced by the membrane's natural composition, especially the ion pairs and the plasticizer's properties. The type of membrane components and quantities were tuned for maximizing the potential responsiveness of the PVC membrane electrode. Both the membrane's three primary components-plasticizers, PVC matrix, and ion-pair association are crucial to the membrane's functionality and sensor response. Because the dielectric constant of the plasticizer affects the potential response, three electrodes were made utilizing various plasticizers, including TBP, BA, and DOP.

Observations led us to the conclusion that the sensors utilizing DOP as a plasticizer were the best ones, as demonstrated in (Table 1). While other elements remained unaltered, the effects of various MBV-TPB association concentrations in the membrane were also investigated. Although the PVC membrane sensor has the best lifespan compared to other sensors, it was discovered that it had an inadequate response. Thus, three electrodes were created, each comprising 10 mg of the ion-pair enhancer, such as α-, β-, and γ-CD. The findings are shown in (Table 1). LOD, Nernstian slopes, and correlation coefficients are computed and presented in (Table 1). The membrane sensor containing 10 mg of β-CD, 8 mg of MBV-TPB, 190 mg of PVC, and 296 mg of DOP was preferred for the following experiments because of its superior performance in terms of slope, correlation coefficient, and LOD (Table 1).

**Table 1:** General characteristics of the different PVC membrane components

MBV-TPB complex (mg)	PVC (mg)	Plasticizer (mg)	Modifiers (mg)	Range (M)	Slope^a^	LOD^a^	Correlation coefficient (*r*)
8	190	315 mg BA	—	1 × 10^−4^ to 1 × 10^−2^	44.2	7.5 × 10^−5^	0.9974
8	190	292 mg TBP	—	1 × 10^−5^ to 1 × 10^−2^	50.3	7.3 × 10^−6^	0.9932
8	190	296 mg DOP	—	1 × 10^−5^ to 1 × 10^−2^	52.7	6.8 × 10^−6^	0.9987
10	190	296 mg DOP	—	1 × 10^−5^ to 1 × 10^−2^	53.6	7.7 × 10^−6^	0.9959
8	190	296 mg DOP	10 mg α CD	1 × 10^−5^ to 1 × 10^−2^	54.4	7.3 × 10^−6^	0.9966
8	190	296 mg DOP	10 mg β CD	1 × 10^−6^ to 1 × 10^−2^	58.7	4.5 × 10^−7^	0.9995
8	190	296 mg DOP	10 mg γ CD	1 × 10^−5^ to 1 × 10^−2^	51.3	6.3 × 10^−6^	0.9991

a Average of three distinct measurements.

#### 3.1.1. pH effect

**Fig. 1:**
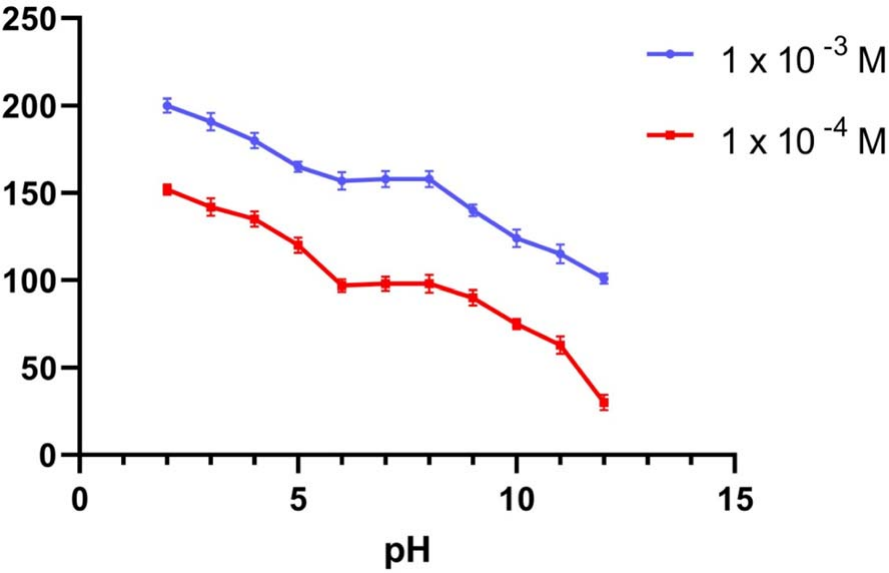
The response of MBV potentiometric to the effect of pH utilizing sensor I.

**Fig. 2:**
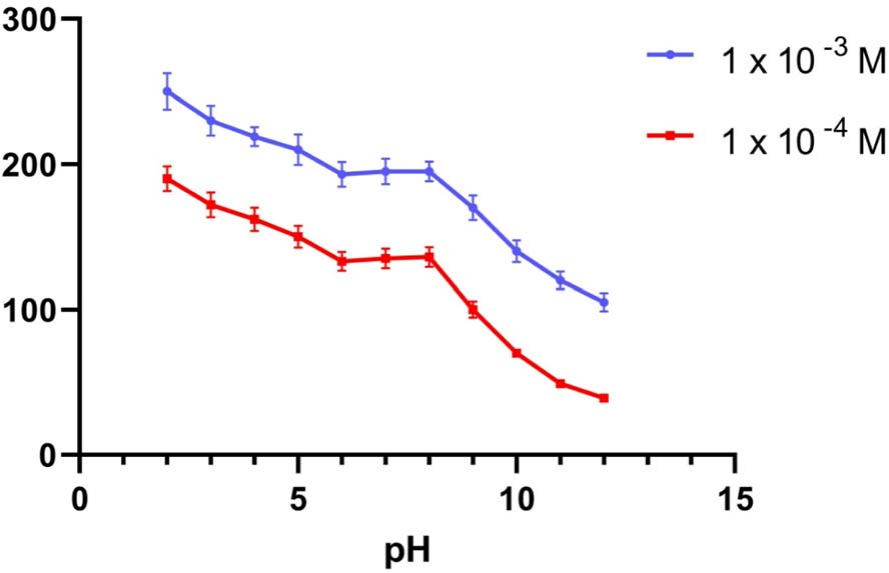
The response of MBV potentiometric to the effect of pH utilizing two middle concentrations (1 × 10^−3^ and 1 × 10^−4^ M) for sensor II.

**Fig. 3:**
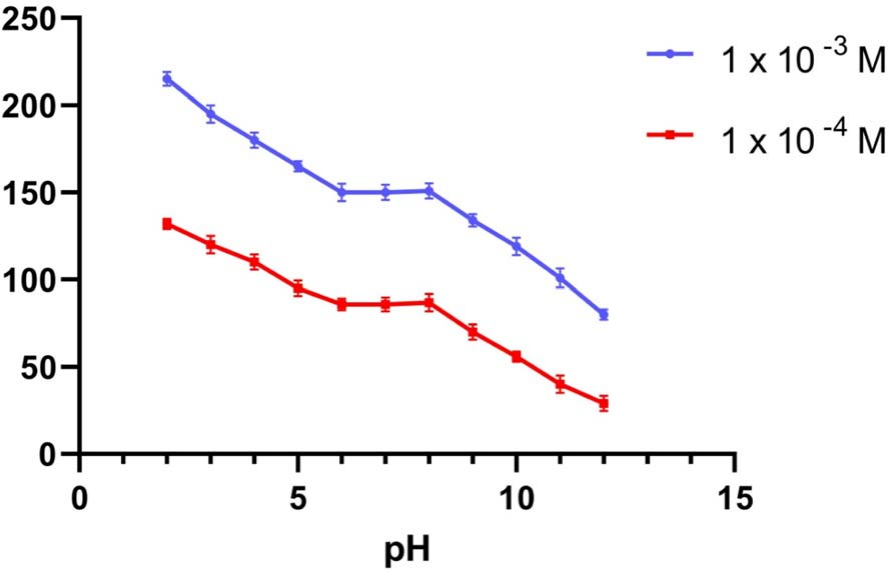
The response of MBV potentiometric to the effect of pH utilizing two middle concentrations (1 × 10^−3^ and 1 × 10^−4^ M) the effect of pH utilizing sensor III.

To provide the perfect trial condition, the impact of pH on various MBV concentrations was investigated at multiple pH ranges (2–12), utilizing BR buffer. The measurement system's pH was adjusted using a 2 M sodium hydroxide solution. The MBV-ion-selective sensors were tested at two concentrations in the middle 0.001 and 0.0001 M concentrations of the MBV solution to represent all concentrations in the linearity range. The pH profile showed that the potential of the tested sensors was constant within the pH range of 6 to 8 (Fig. 1–3). Because the concentration of the un-protonated forms of MBV steadily increased at higher pH levels (pH > 8), the potential decreased. The pH range of 6 to 8 was subsequently considered to be the best pH range for the three sensors.

#### 3.1.2. Soaking effect in MBV solutions

It has the positive effect of producing a gel layer at the modified membrane surface where ion exchange occurs, the freshly manufactured sensors were immersed in a 0.01 M MBV solution for a fixed time, and different periods (0–36 h) were examined. Potential measurements *vs.* negative logarithmic AMP concentration values were plotted to establish calibration curves, and the slopes were then computed. The optimum soaking time, which will give the best results for analyzing MBV using three sensors was 24 h (Table 2).

**Table 2:** Soaking time impact in MBV on the behaviour of the manufactured sensors I, II, and III

Soaking time/h	Slope		
	Sensor I	Sensor II	Sensor III
0	49	50.8	44
2	51.6	51.1	45.3
4	51.9	51.8	48.5
6	52.3	51.6	48.1
12	52.7	53.4	49.3
20	54.1	57.9	50.6
24	54.4	58.7	51.3
30	54.00	57.9	51.2
36	52.6	57.6	44.5

#### 3.1.3. Dynamic response time

**Fig. 4:**
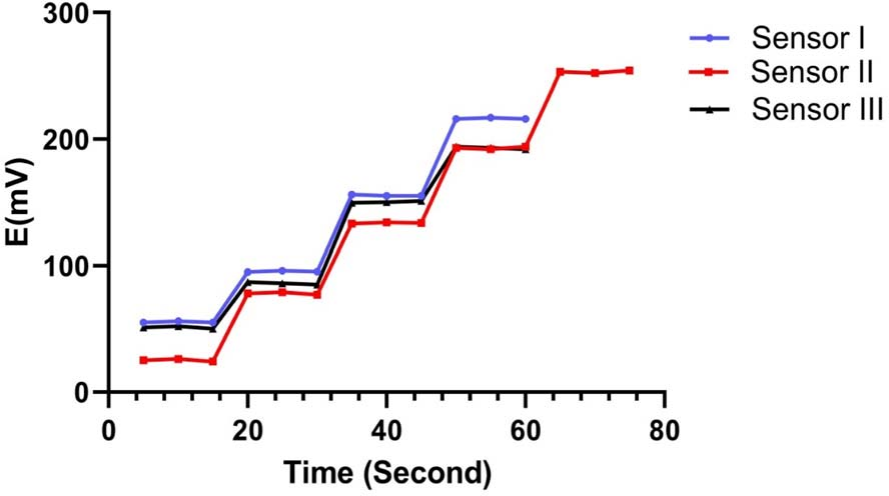
The response time curve of MBV using concentrations of 10^−5^ to 10^−2^ for both sensors 1, 3 and concentrations 10^−6^ to 10^−2^ for sensor 2.

The dynamic response times of the constructed sensors were evaluated by monitoring the time necessary to establish a steady state potential (±1 mV) following a rapid rise in the MBV concentration from 1 × 10^−7^ to 1 × 10^−2^ M. Fig. 4 shows that the time needed for sensors I, II and III was 5 seconds (Table 3).

**Table 3:** The electrochemical characteristic responses for three fabricated MBV sensors

Parameter		Sensor I	Sensor II	Sensor III
Concentration range (M)		1 × 10^−5^ to 1 × 10^−2^	1 × 10^−6^ to 1 × 10^−2^	1 × 10^−5^ to 1 × 10^−2^
Slope (mV per decade)		54.4	58.7	51.3
Intercept (mV)		320.9	369.0	296.7
Correlation coefficient (*r*)		0.9966	0.9995	0.9991
Accuracy (recovery% ± S.D.)		100.77 ± 0.543	98.33 ± 0.675	101.02 ± 0.231
LOD (M)		7.3 × 10^−6^	4.5 × 10^−7^	6.3 × 10^−6^
Time of response (s)		5 ± 0.1	5 ± 0.1	5 ± 0.1
Working pH range		6–8	6–8	6–8
Stability (weeks)		4	4	4
Reproducibility (% RSD)		1.88	1.55	1.98
Precision (% RSD)	Intra-day	0.709	0.926	1.035
	Inter-day	1.089	1.433	1.709
Robustness (mean ± % RSD)		100.12 ± 0.926	99.16 ± 1.591	99.67 ± 1.313

#### 3.1.4. Lifetime

The electrochemical performance of the three recommended sensors was evaluated using the data from IUPAC recommendations. The sensors were tested following the procedure described in the calibration curves section, and at the specified time intervals, the results of linear range, slope, and limit of detection of these electrodes were recorded. The PVC polymeric membrane could withstand the soaking effect for up to 4 weeks, according to a study conducted over several weeks.

#### 3.1.5. Selectivity of the electrode

The responses of the mebeverine sensors to a variety of organic and inorganic particles were examined. Using a separate solution or mixed solution approach, the potentiometric selectivity coefficients were calculated in accordance with IUPAC recommendations using a BR buffer of pH 7. Table 4 summarizes the findings of the selectivity coefficient. The results shown in Table 4 revealed that the suggested approach was free from influence from the studied ions due to its low selectivity coefficient.

**Table 4:** Selectivity coefficients of the MPM and SSM-based sensors under investigation

Interfering ions^a^	Sensor I	Sensor II	Sensor III
***K*** ^**pot**^_**drug JZ**_ **SSM** b			
KCl	3.88 × 10^−4^	1.17 × 10^−3^	4.67 × 10^−3^
CaCl_2_	4.66 × 10^−4^	2.67 × 10^−4^	6.32 × 10^−4^
MgCl_2_	5.35 × 10^−4^	2.01 × 10^−4^	3.67 × 10^−4^
NaCl	6.46 × 10^−3^	2.12 × 10^−3^	3.09 × 10^−4^
NH_4_Cl	2.89 × 10^−4^	3.14 × 10^−2^	4.56 × 10^−4^
NiCl_2_	5.95 × 10^−3^	6.17 × 10^−2^	2.33 × 10^−2^
BaCl_2_	8.23 × 10^−3^	4.45 × 10^−2^	2.56 × 10^−3^

**MPM** c			
Glucose	4.12 × 10^−2^	1.78 × 10^−4^	3.15 × 10^−2^
Urea	5.17 × 10^−2^	2.23 × 10^−4^	1.12 × 10^−3^
Glycine	2.89 × 10^−2^	4.15 × 10^−3^	3.77 × 10^−2^
Sucrose	7.13 × 10^−2^	3.33 × 10^−4^	2.11 × 10^−2^
Starch	6.16 × 10^−2^	2.60 × 10^−3^	4.32 × 10^−3^
Lactose	1.33 × 10^−3^	4.12 × 10^−3^	2.90 × 10^−3^

a Interfering ion concentration (1 × 10^−3^ M).

b Separate solution method.

c Matched potential method.

#### 3.1.6. Molecular docking (MD)

**Fig. 5:**
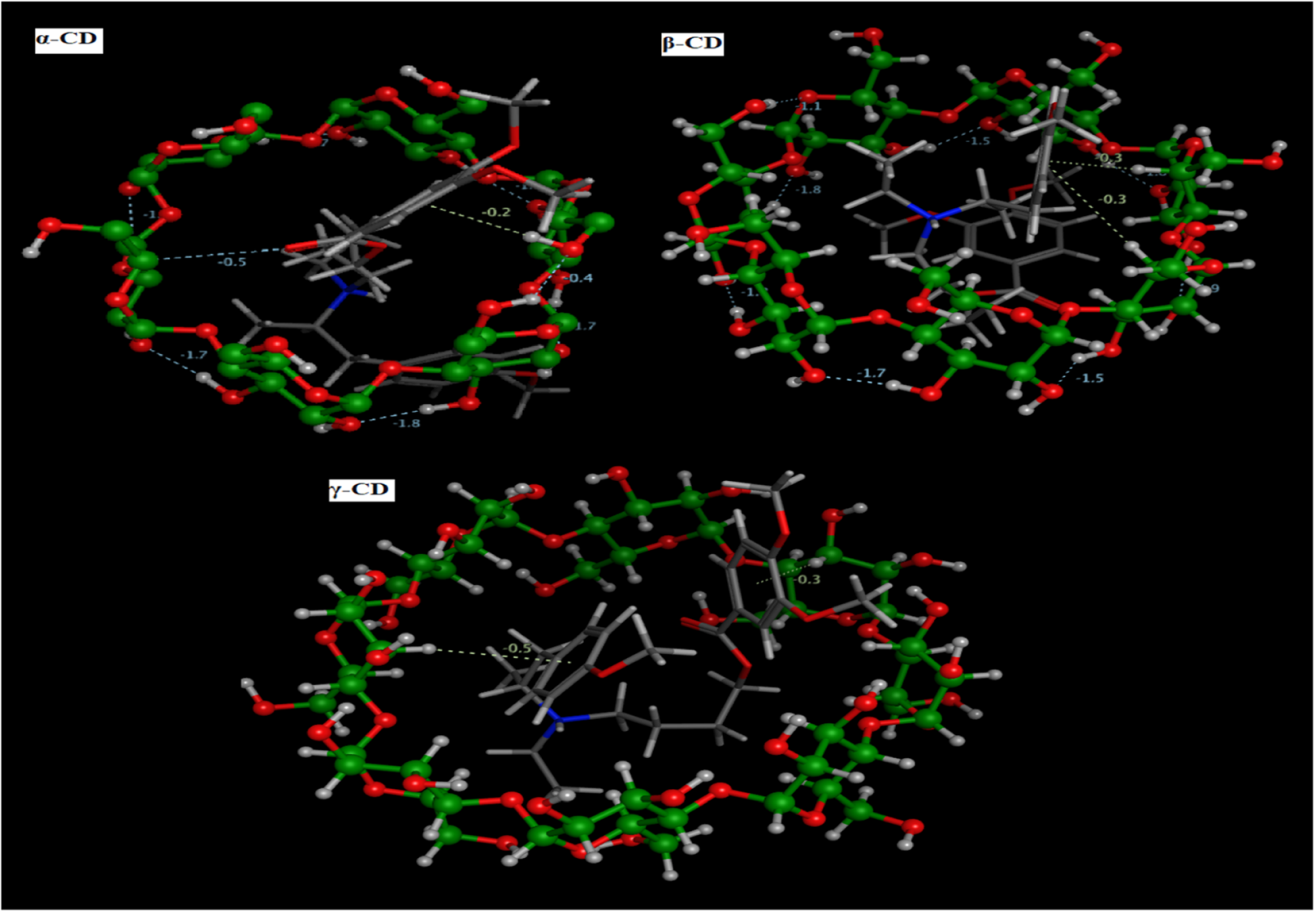
MBV 3D molecular interactions through (α, β and γ) CD pockets.

**Fig. 6:**
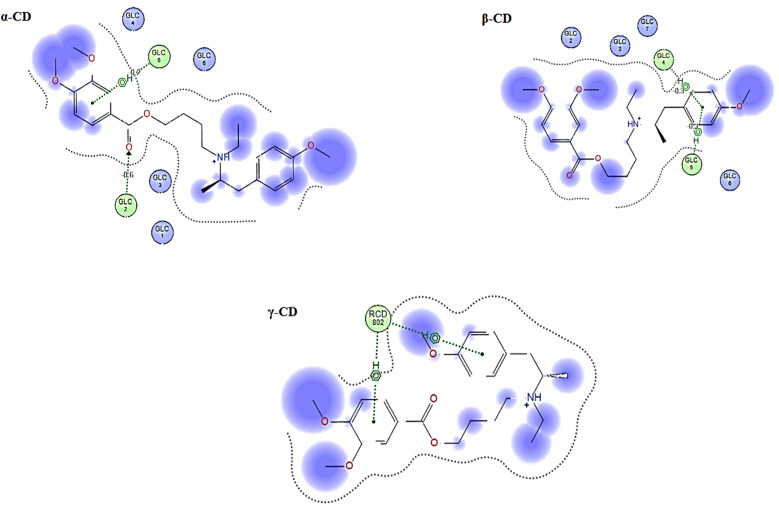
The guest–host 2D interaction plot displaying MBV's binding site with ionophores (α, β and γ) CD ion pair modifiers.

**Fig. 7:**
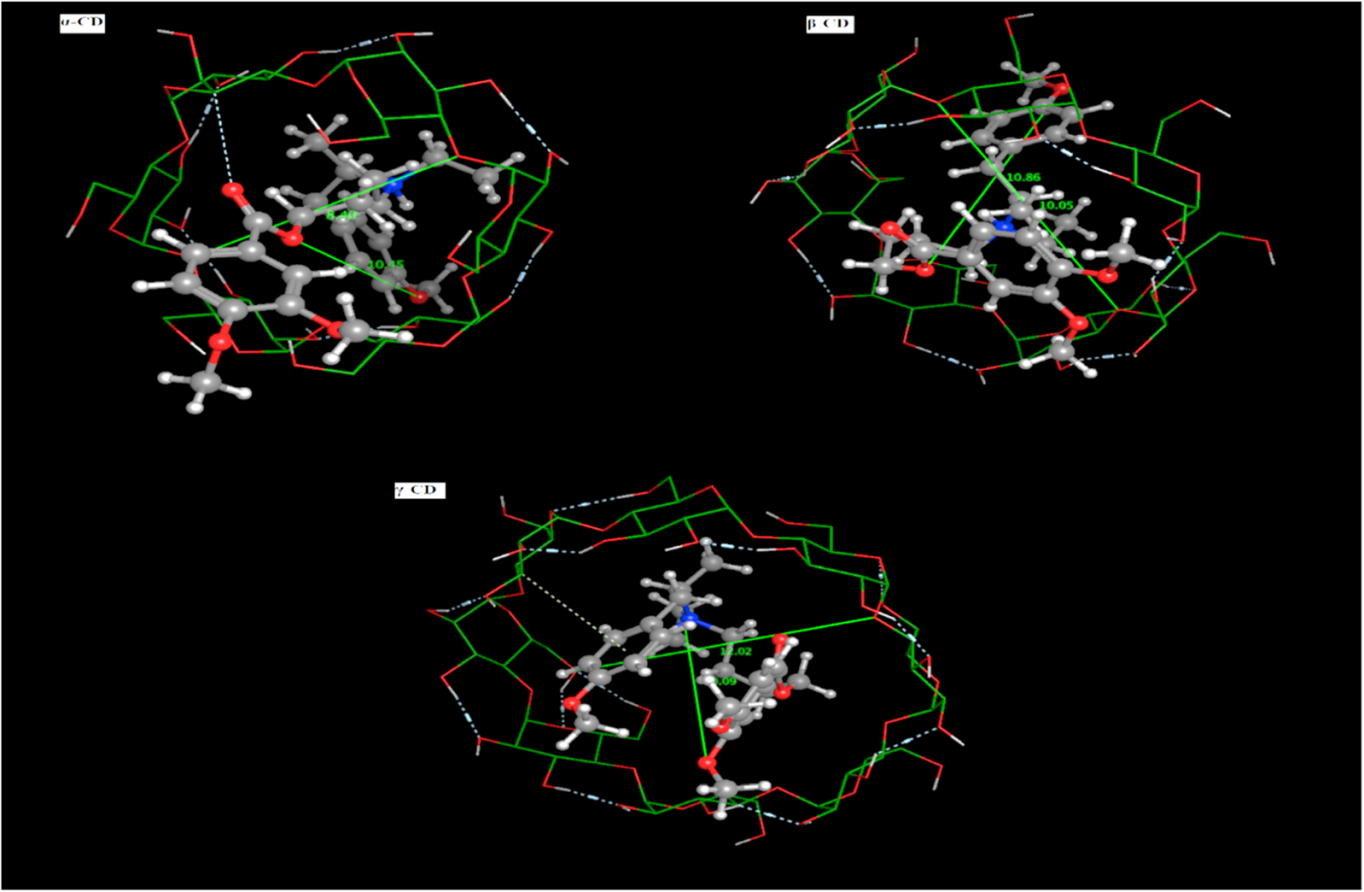
Mebeverine HCl molecular diameters with (α, β and γ) CD ion pair modifiers.

The prediction of the interaction of a host (CD) and guest molecule (MBV), orientation, and molecular fitting was acquired using the MD process. The purpose of this study was to confirm which sensor is the best to measure the MBV by determining the optimal electrode modification. Based on our experimental investigations, MBV showed substantial interactions with both (α, β, and γ) CD. The potentiometric response shows a monovalent Nernstian response for the constructed CD-based sensors and was used to observe these interactions. According to MD findings (Fig. 5), which were matched to the potentiometric slopes of the constructed sensors (54.4, 58.7, and 51.3 mV per decade, respectively), the docking scores for both (α, β, and γ) CD were found to be −6.25, −9.12, and −8.53 kcal mol^−1^ (Fig. 6). shows the optimal docking of MBV with α, β, and γ-CD *via* the electrostatic interactions, dipole–dipole interactions, van der Waals forces, and hydrogen bonding. Furthermore, this figure shows that β-CD explores strong binding affinity and an intense van der Waals force with MBV (−1.7, −1.5, and −0.3 kcal mol^−1^) when compared to α- and γ-CD. The host (CD) and guest (MBV) molecular diameters revealed that the average diameter for MBV was 10.5 A, whereas, the average diameters for α, β, and γ-CD were 8.40, 10.05, and 12.02 A, respectively, (Fig. 7). β-CD ionophore is the most suited diameter for perfect inclusion with MBV molecule (Fig. 7), leading to the high stability of the formed complex, which corresponds to the docking scores above.

### 3.2. Method validation

The presented method was validated as per the IUPAC recommendations, and the proposed sensor assays have been validated including linearity and range, the limit of detection, robustness, accuracy, and precision.^30^

#### 3.2.1. Linearity and range

**Fig. 8:**
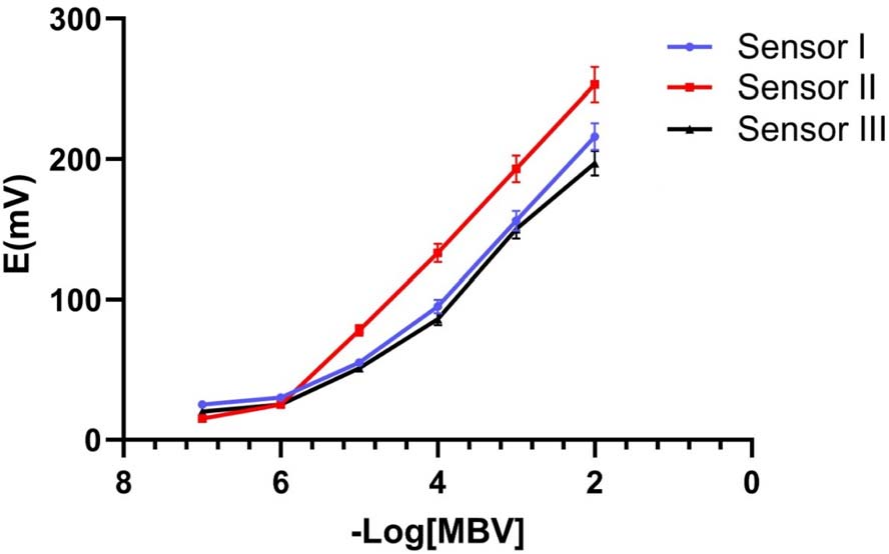
Potential calibration profile for the proposed sensors I, II, and III.

The linearity of the analytical method is defined as the concentration range where the acquired findings are directly related to the quantity of analyte in the sample. It was demonstrated that sensor II had a Nernstian response spanning concentration ranges of 1 × 10^−6^ to 1 × 10^−2^ M. Where sensors I and III had a response over the concentration range of 1 × 10^−5^ to 1 × 10^−2^ M as presented in Fig. 8. The correlation coefficients for sensors I, II, and III were calculated and found to be 0.9966, 0.9995, and 0.9991, respectively (Table 3).

#### 3.2.2. Accuracy

The recovery of MBV was calculated by comparing the purposeful concentration with that discovered by the direct inclusion method in the Britton–Robinson buffer of pH 7, using three various concentrations of MBV solution. The recovery percentage at each concentration was recorded using the given mathematical expression (measured concentration/actual concentration) × 100. Table 3 shows the average recoveries of the three sensors with their S.D.

#### 3.2.3. Precision

The intra- and inter-day precisions of the analysis were tested on that day and the other three different days by analyzing three replicates of three different MBV concentrations. RSD% was used to account for the precision. The results obtained (Table 3) were within the acceptable range of less than 2%. Moreover, reproducibility is a parameter for testing measurements that was investigated for the three sensors and it was found that the % RSD of five runs at five similarly prepared proposed electrodes was calculated to be less than 2% values, which revealed a good reproducibility of the proposed electrodes.

#### 3.2.4. Robustness

To study the method's robustness, the trial conditions that influenced the potential response, such as soaking time and pH, were investigated. A preliminary analysis of the data under these various settings suggested that the method was completely robust and that the primary pH variable should fall within the range of 6 to 8, where the ideal pH was 7, employing a BR buffer (Table 3).

#### 3.2.5. The limit of detection (LOD)

The lowest amount of the substance under investigation that could be identified in a sample is known as the limit of detection (LOD). The LOD values shown in Table 3 indicate that the fabricated sensors could detect very low MBV concentrations, allowing them to extend MBV quantitation to pharmaceutical formulation.

### 3.3. Potentiometric analysis of MBV in pharmaceutical preparation

The presented MBV sensors were used to measure MBV in Colospasmin® forte tablets without the need for prior drug derivatization or extraction, as shown in Table 5. The direct method evaluation showed that the assay's accuracy and precision are shown by the excellent percentage recoveries (% *R*) of MBV in the pharmaceutical form, which ranged from 99.29 to 100.12%, with the S.D. range from 0.867 to 1.193. In order to evaluate the method's accuracy, the standard addition procedure was used. The results are shown in Table 6.

**Table 5:** Statistical comparison of the potentiometric approaches that have been established with those that have been previously reported

Item	Reported method^21^	Sensor I	Sensor II	Sensor III
Mean	100.27	99.99	99.29	100.12
S.D.	1.193	0.943	0.888	0.867
*n*	5	5	5	5
Variance	1.423	0.891	0.788	0.752
Student's *t* test^a^	—	1.34 (2.31)	1.33 (2.31)	1.12 (2.31)
*F*-test^a^	—	1.597 (6.338)	1.805 (6.338)	1.890 (6.338)

a The values in parenthesis are the corresponding theoretical values for *t* and *F* at *P* = 0.05.

**Table 6:** Application of standard addition techniques for MBV analysis

Standard addition technique (M)		Sensor I		Sensor II		Sensor III	
Pure added ×10^−4^ M	Tablet taken M	Pure found ×10^−4^ M	% found	Pure found ×10^−4^ M	% found	Pure found ×10^−4^ M	% found
1	10^−3^	0.987	98.70	1.012	101.2	1.012	101.2
2		2.03	101.5	1.97	98.50	2.02	101.00
3		3.02	100.66	3.01	100.33	2.91	97.00
Mean^a^			100.29		100.01		99.73
S.D.			1.436		1.378		2.369

a Three measurements mean.

#### 3.3.1. Statistical analysis

By comparing the outcomes of the newly discovered techniques to those attained by the previously published method, satisfactory findings were obtained. The student's and *F*-ratio tests revealed outstanding consistency between the reported techniques and the developed methods. The results of the two tests were found to differ insignificantly, and all findings are compiled in Table 5. The recovery percentages findings of the pharmaceutical preparation obtained from the new techniques and described method were compared using one way-ANOVA. No significant differences were observed (Table 7). These findings attest to the validity of applying the developed techniques to estimate MBV in pharmaceutical preparations.

**Table 7:** One-way ANOVA on recovery percentage data acquired from the application of potentiometric methods as a statistical analysis at 95% confidence interval

MBV					
Variation source	Sum of squares	Degree of freedom	Mean square	*F*-value	*P*-value
Between groups	2.788	3	0.929	0.964	0.433
Within groups	15.422	16	0.963		
Total	18.211				

## 4. Conclusions

The current study displayed three variously constructed CD-based MBV potentiometric sensors. With a quick response time (5 s) and a long lifespan, the potentiometric techniques demonstrated that the optimum Nernstian slope was obtained from the β-CD sensor in the concentration range of 10^−6^ to 10^−2^ M. The obtained information was supported by molecular docking research, which demonstrated that β-CD had the most outstanding docking score. As a result of having the most considerable binding energy, the MBV-CD complex was the most stable. We may conclude that the β-CD sensor is very accurate and precise for the quantitative assessment of MBV in the pure and its dosage form and employed in regular analysis for drug quality control. Additionally, MD was implemented to assess the ability of the proposed method to meet the specification limits and confirm the experimental work.

## Conflicts of interest

The authors have no conflicts of interest to declare.

## Supplementary Material
